# Combining bacteriophage engineering and linear dichroism spectroscopy to produce a DNA hybridisation assay†

**DOI:** 10.1039/d0cb00135j

**Published:** 2020-10-23

**Authors:** Aysha Ali, Haydn A. Little, Jake G. Carter, Craig Douglas, Matthew R. Hicks, David M. Kenyon, Christophe Lacomme, Richard T. Logan, Timothy R. Dafforn, James H. R. Tucker

**Affiliations:** School of Chemistry, University of Birmingham Edgbaston Birmingham B15 2TT UK j.tucker@bham.ac.uk; School of Biosciences, University of Birmingham Edgbaston Birmingham B15 2TT UK t.r.dafforn@bham.ac.uk; SASA Roddinglaw Road Edinburgh EH12 9FJ UK; Linear Diagnostics Ltd 97 Vincent Drive Birmingham B15 2SQ UK

## Abstract

Nucleic acid detection is an important part of our bio-detection arsenal, with the COVID-19 pandemic clearly demonstrating the importance to healthcare of rapid and efficient detection of specific pathogenic sequences. As part of the drive to establish new DNA detection methodologies and signal read-outs, here we show how linear dichroism (LD) spectroscopy can be used to produce a rapid and modular detection system for detecting quantities of DNA from both bacterial and viral pathogens. The LD sensing method exploits changes in fluid alignment of bionanoparticles (bacteriophage M13) engineered with DNA stands covalently attached to their surfaces, with the read-out signal induced by the formation of complementary duplexes between DNA targets and two M13 bionanoparticles. This new sandwich assay can detect pathogenic material down to picomolar levels in under 1 minute without amplification, as demonstrated by the successful sensing of DNA sequences from a plant virus (*Potato virus Y*) and an ampicillin resistance gene, *ampR*.

## Abstract 2

A novel DNA sensing method based on LD spectroscopy and using bionanoparticle scaffolds is described, as demonstrated by the rapid detection of DNA strands associated with bacterial and viral pathogens.

## Graphical abstract

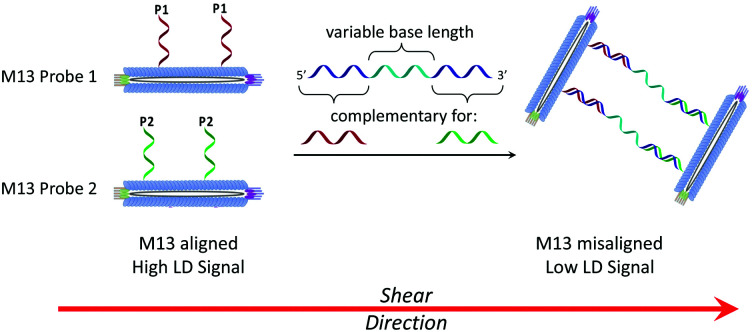

## Introduction

Effective nucleic acid (DNA or RNA) detection is essential for flagging and monitoring genetic diseases, as well as identifying dangerous pathogens. Despite ongoing advances in sequencing technologies, the most widely used nucleic acid detection methods, commercial or otherwise, continue to involve fluorescence spectroscopy. This can largely be attributed to ubiquitous real-time polymerase chain reaction (PCR) assays (*e.g.* as used in SARS-CoV-2 detection for COVID-19 diagnosis), in which fluorescent molecules denote the presence of DNA.^1^ These and related approaches often involve fluorophore-tagged oligomer probes,^2^ whose binding (hybridization) to target DNA results in a transducible signal.

The continued focus on fluorescence-based systems has meant that technical advances are now reducing in frequency and impact as assays move ever closer to the optimum. To address this slowed rate of development, other methods for DNA detection based on hybridization have emerged, including electrochemical,^3^ electronic^4^ and other spectroscopic^5^ techniques. To achieve sensitivity, some of these sensor systems are also used in tandem with various DNA amplification methods such as PCR to generate sufficient levels of target and hence, sensitivity for the test. While these new methods give a wide array of options for DNA detection, concerns such as complexity, cost, lack of portability and slow detection times continue to present hurdles to their commercial adoption.^6^ Here we demonstrate the development of a new DNA sensing technique, based on linear dichroism (LD) spectroscopy, as a potential solution to some of these issues.

Shear flow aligned LD is a method that provides an optical signal whose intensity is proportional to the alignment of chromophores in fluid flow. The M13 bacteriophage is a viral nanoparticle that is cheap to produce, being readily harvested from *E. coli*. Furthermore, its dimensions make it readily alignable in flow, being *ca.* 1 micron long and less than 10 nm wide. This means that under shear flow conditions, intrinsic M13 chromophores (*e.g.* amino acid residues on its protein coat and nucleobases in its genome) absorb strongly when subjected to linearly polarised light. In previous M13 conjugation experiments,^7,8^ we demonstrated that by attaching antibodies or DNA primers to the coat of the M13, LD sensors for bacteria (*E. coli*)^7*a*^ or genes^8^ could respectively be generated. This was a result of M13 alignment in shear flow being altered by target binding, which in turn altered the LD signal, providing a concentration dependent read-out for the presence of the analyte.

**Scheme 1:**
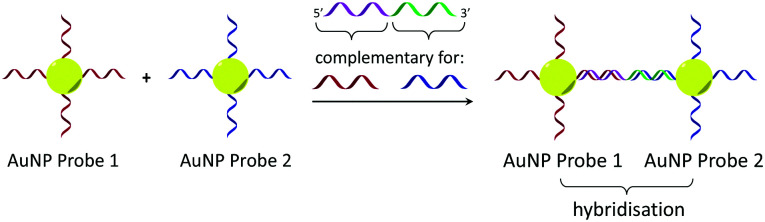
Gold nanoparticle sandwich assay described by Mirkin *et al.*^5*e*^ The left-hand side shows DNA-modified AuNPs in the absence of the DNA target. The right-hand side shows their aggregation in the presence of complementary DNA, which gives rise to a change in absorbance (SPR band) in the visible spectrum.

**Scheme 2:**
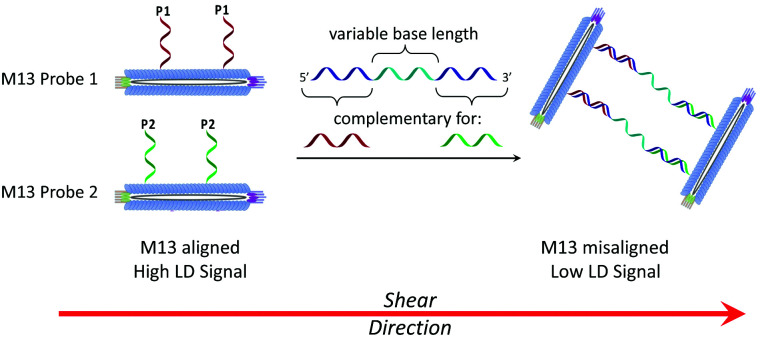
The bionanoparticle sandwich assay in this work. The left-hand side shows DNA-modified M13 bacteriophages that are aligned in shear flow in the absence of the DNA target. The right-hand side shows their aggregation in the presence of complementary DNA, which causes misalignment and a reduction in the LD signal.

In considering the further development of our LD-based sensing methodology to make it more applicable to DNA strand detection, we next considered the “sandwich” assay. This is a popular DNA sensing technique^9^ due to the ease in which separate probes can be designed to bind specifically to different sequences within a single target, providing a high degree of selectivity. Mirkin *et al.*, were the first to demonstrate an optical-based DNA sandwich assay based on the aggregation of gold nanoparticles (Scheme 1).^5*e*^ We considered that using this methodology with M13 bionanoparticles would improve not only target selectivity but also LD signal sensitivity, as binding would be expected to trigger significant changes in alignment due to M13 aggregation (Scheme 2). This would allow much smaller pieces of DNA to be detected and provide a more modular design, with the assay placed at the end of a wide range of DNA amplification techniques. Herein, we report the first example of such an LD-based sandwich assay and demonstrate its high adaptability and modularity, with the rapid (<1 min) detection of DNA from two different types of pathogen: (i) an antibiotic-resistant bacterium and (ii) a common potato virus, with limits of detection (LODs) down to the picomolar range.

## Results and discussion

### Assay design and probe preparation

Previously we established the chemical method whereby DNA oligomers are attached to the pVIII protein on the surface of the M13 bacteriophage to give an M13–DNA conjugate.^8^ Our new sandwich assay would involve the production of two DNA–M13 conjugates, with one complementary to the 5′ end of the target and the other complementary to the 3′ end, as shown in Scheme 2. The hypothesis was that in the absence of target, both conjugates would be able to align in shear flow and hence produce an LD signal. However, in the presence of the target, the two types of conjugate would be “bridged” by the target DNA. The presence of multiple DNA probes on each conjugate and hence the formation of many “bridges” would lead to the formation of an aggregate. This aggregate would significantly perturb the hydrodynamics of the conjugates, reducing alignment in shear flow, which would consequently generate the LD sensing signal.

To develop this new assay format, a number of sequential steps were required. Firstly, the DNA probe sequences had to be designed so that they could bind to the complementary sequences of the target DNA. Next these strands were to be conjugated to the M13 coat protein using the methods that we established previously.^8^ Then the ability of these modified bionanoparticles to bind DNA targets would be tested using fluorescence anisotropy. Finally, the scope of this new LD sensing approach would be assessed by targeting DNA from both bacterial and viral pathogens.

Table 1 lists the various targets used in this study as well as the corresponding M13 probes. Target strand **PVY-Target 1** is a 26-mer cDNA sequence of the coat protein gene of the potato-infecting virus species, *Potato virus Y* (PVY), a common target in PCR-based detection methods for plant pathogens.^10^ Target strand **AMPR-Target 1** is a 39-mer sequence from the ampicillin resistance gene, *ampR*, which we studied previously.^8^ We also decided to assess longer targets with additional thymine bases (**AMPR-Target 2** and **AMPR-Target 3**, with runs of 10 and 20 Ts respectively) to investigate the effect on the LD signal of having a more flexible target with non-binding regions. The viral probes **PVY-1** and **PVY-2** each contained a polyT linker to ensure that the attached strands had sufficient clearance from the M13 coat for effective binding of their relatively small 26-mer target. All four M13 probes (two for each target) contained visible fluorophore dyes (either rhodamine or fluorescein based) to facilitate their purification and characterisation, and also to enable the use of anisotropy measurements to confirm DNA duplex formation. Previous studies demonstrated that attaching fluorophores to the M13 bacteriophage gave no discernible change in alignment in flow.^7*b*^ The two stage method of conjugating the precursor DNA strands to lysine residues on the pVIII protein of the M13, and their subsequent purification by size-exclusion chromatography, were carried out as described previously (see ESI† for further information).^8^ In the case of the bacterial probes, the average number of DNA strands per M13 nanoparticle was calculated to be 15, giving a conjugation efficiency of 15%, almost double the value reported previously for **AMPR-P1**.^8^

**Table 1:** M13 probes and their target sequences

Name	Sequence (5′–3′)
**AMPR-P1**	M13-S-(TAMRA-dT)-ATG AGT ATT CAA CAT TTC
**AMPR-P2**	GCC TCA CTG ATT AAG CAT TGG-(6-FAM)-S-M13
**AMPR-Target 1**	CCA ATG CTT AAT CAG TGA GGC GAA ATG TTG AAT ACT CAT
**AMPR-Target 2**	CCA ATG CTT AAT CAG TGA GGC TTTTTTTTTT GAA ATG TTG AAT ACT CAT
**AMPR-Target 3**	CCA ATG CTT AAT CAG TGA GGC TTTTTTTTTTTTTTTTTTTT GAA ATG TTG AAT ACT CAT
**AMPR-NC**	ATG AGT ATT CAA CAT TTC GCC TCA CTG AAT AAG CAT TGG
**PVY-P1**	M13-S-(6-FAM)-TTT TTT TTT GAA AAT GGA ACC
**PVY-P2**	TCG CCA AAT GTC ATT TTT TTT T-(6-FAM)-S-M13
**PVY-Target 1**	TGA CAT TTG GCG AGG TTC CAT TTT CA
**PVY-NC**	TGA AAA TGG AAC CTC GCC AAA TGT CA

### Fluorescence anisotropy studies

**Fig. 1:**
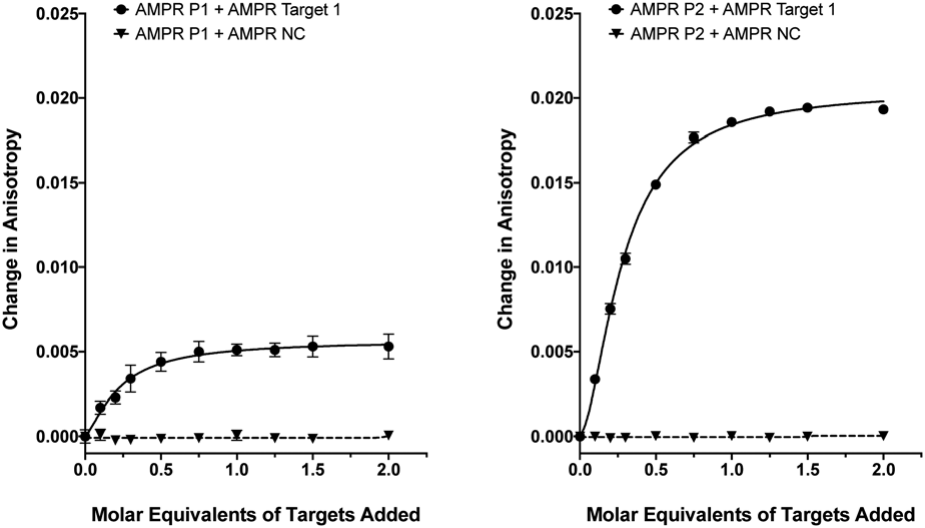
Fluorescence anisotropy changes of M13 probes **AMPR-P1** and **AMPR-P2** (each phage at 1.4 nM) upon the addition of **AMPR-Target 1** (circles) and **AMPR-NC** (triangles) shown as DNA molar equivalents, 150 mM NaCl, 100 mM potassium phosphate buffer, pH 7.2, RT. Data shows *n* = 3 (SEM).

As we have previously shown,^8^ the fluorescence anisotropies of fluorescent dyes are known to be sensitive to the molecular motions within DNA strands, with increases in anisotropy indicating increased probe rigidity and/or longer tumbling times in solution. These changes can result from interaction with the complementary strand, providing a simple assay for duplex formation. To test the ability of the DNA-tagged M13 systems to bind their targets, the two bacterial probes (**AMPR-P1** and **AMPR-P2**) were each mixed with **AMPR-Target 1**, and the anisotropy measured. In each case, increasing concentrations of target led to an increase in fluorescence anisotropy, with no changes observed for the non-complementary control strand (Fig. 1). As expected, the increase in signal plateaued out at a 1 : 1 DNA : DNA stoichiometry, where one DNA molar equivalent corresponds to 15 molar equivalents of target per M13 bionanoparticle. Similar changes were observed for the unconjugated probes with the same target (see ESI†), which gave further evidence for these changes arising due to duplex formation, as well as indicating that conjugation of DNA to the surface of the bacterio-phage had no adverse effect on duplex formation.

### LD studies – sensing of pathogenic ssDNA

Our previous studies revealed that conjugation of DNA to the coat protein of the M13 bacteriophage to form **AMPR-P1** resulted in only minor changes to LD spectrum in the region where the bacteriophage groups absorb light.^8^ This was backed up by further studies on the two viral probes (see ESI†), which revealed only a small decrease in the LD signal compared to unfunctionalised wild-type M13 (wtM13).

**Fig. 2:**
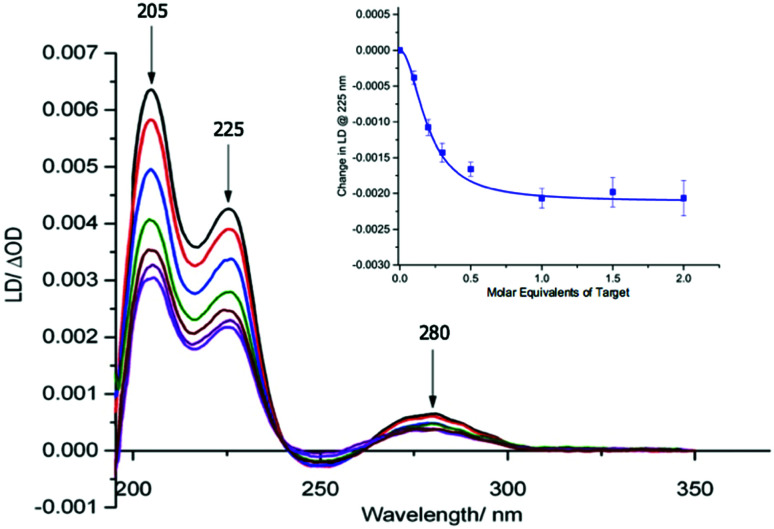
Overlaid LD spectra showing the effect of adding increasing amounts of **AMPR-Target 1** to M13 probes **AMPR-P1** and **AMPR-P2** (each phage at 1.4 nM), 150 mM NaCl, 100 mM potassium phosphate buffer, pH 7.2, RT, sample volume 100 μL. Inset: Change in LD signal (ΔOD) at 225 nm plotted against DNA molar equivalents of **AMPR-Target 1**. Data shows *n* = 3 (SEM).

With retention of the LD signal upon DNA conjugation confirmed, indicating alignment of the modified bionanoparticles in shear flow, the next task was to establish whether the chosen bacterial and viral pathogenic targets could be sensed in a DNA sandwich assay. A series of experiments were therefore undertaken involving the addition of aliquots of complementary and non-complementary targets to the probes in various combinations. The data for bacterial **AMPR-P1** and **AMPR-P2** together in the presence of increasing amounts of **AMPR-Target 1** is shown in Fig. 2. An overall decrease in the LD signal was indeed observed (within 1 min of each addition), indicating that the hydrodynamics of the DNA–M13 particles were instantly altered, reducing their alignment in shear flow. By monitoring the LD signal at 225 nm (Fig. 2 inset) we were able to confirm that M13-probe saturation occurred upon the addition of approximately an equimolar (DNA:DNA) amount of **AMPR-Target 1**. Similar changes were observed for the viral probe system, for which a limit of detection (LOD) study showed that the **PVY-Target 1** could be detected at 50 pM without amplification (see ESI†). This is comparable to the reported LODs of methods to detect nucleic acids such as the lateral flow assay (60 pM sensitivity)^11^ and some other optical hybridization methods (2 nM sensitivity).^12^

**Fig. 3:**
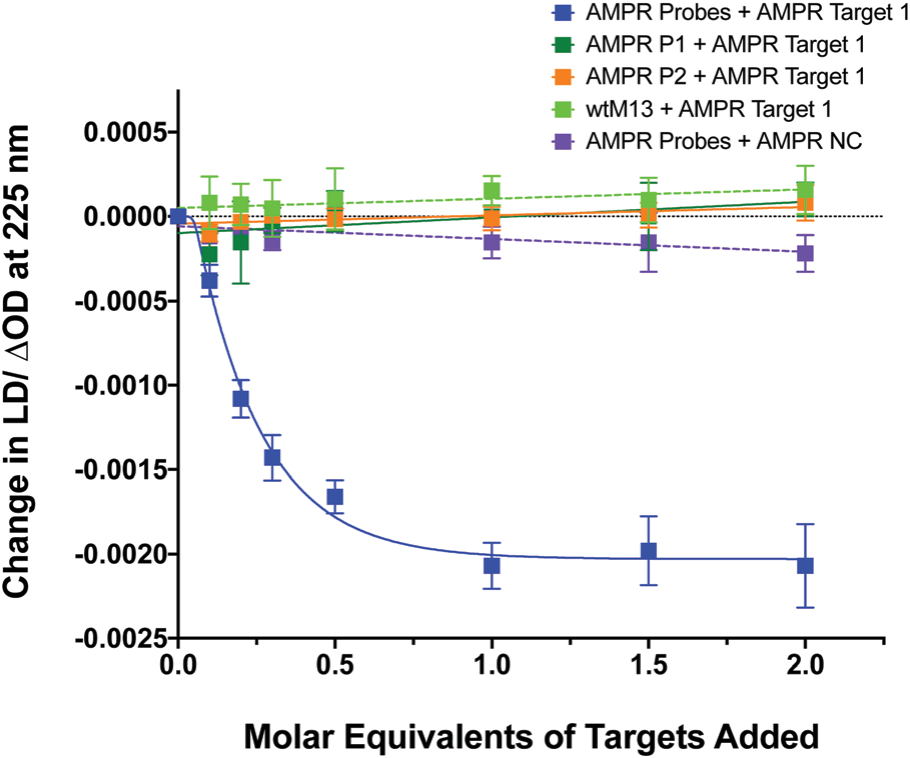
Control experiments showing the effect on the LD signal at 225 nm of adding **AMPR-Target 1** or **AMPR-NC** to various combinations of M13 probes **AMPR-P1** and **AMPR-P2** or unfunctionalised phage, wtM13 (each phage at 1.4 nM, DNA molar equivalents shown), 150 mM NaCl, 100 mM potassium phosphate buffer, pH 7.2, RT, sample volume 100 μL. Data shows *n* = 3 (SEM).

To confirm that the change in LD signal was the result of DNA:DNA binding consistent with a sandwich type interaction, a series of control experiments were carried out on the bacterial system using both unfunctionalised M13 (wtM13) and DNA targets lacking complementary sequences (Fig. 3). In all these cases no significant change in LD signal was observed, with a large decrease observed only for the situation in which both probes were present. This indicates that the target must be bound by both probes to affect the LD signal, which would be consistent with the desired sandwich-type interaction increasing particle aggregation. When only one probe was present, essentially the same effect as a non-specific sequence was observed, with duplex formation unable to link multiple M13 moieties together.

The reversibility of the system was readily demonstrated by heating the aggregated M13–DNA assemblies to 65 °C (*i.e.* to approximately the melting temperatures of the duplexes), which regenerated the original LD signal (see ESI† for further information). Cooling the samples to RT regenerated the sensing signal, clearly demonstrating not only the binding-mediated dependence of the technique but also its thermal robustness.

**Fig. 4:**
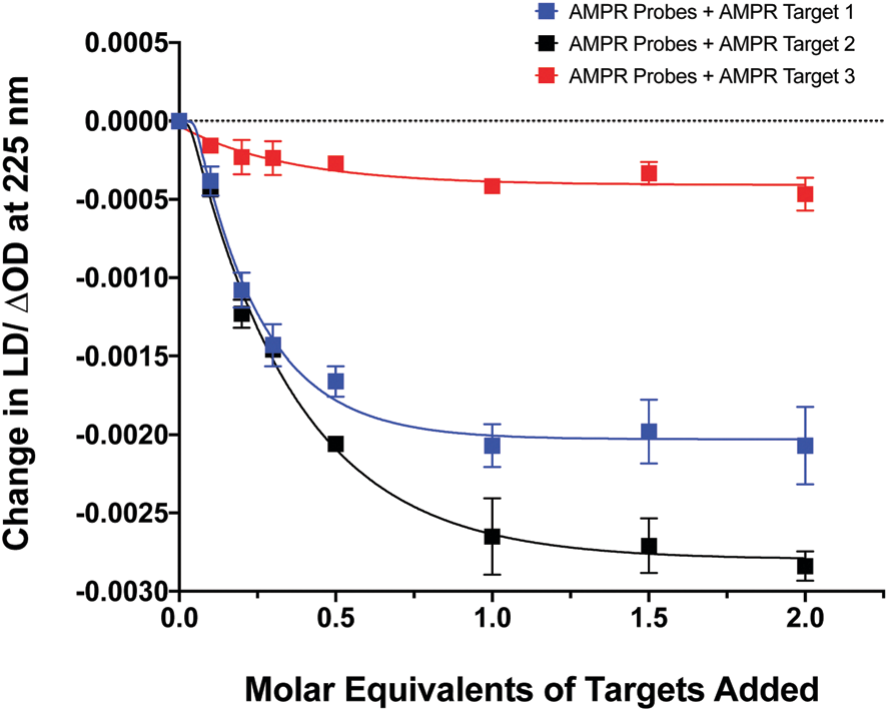
Change in LD signal at 225 nm plotted against DNA molar equivalents of three different targets to M13 probes **AMPR-P1** and **AMPR-P2** (each phage at 1.4 nM), 150 mM NaCl, 100 mM potassium phosphate buffer, pH 7.2, RT, sample volume 100 μL. Data shows *n* = 3 (SEM).

The influence of the distance between the complementary sequences in the bacterial target on the reduction in LD signal by comparing the addition of **AMPR-Target 1** with that of **AMPR-Target 2** and **AMPR-Target 3** (Fig. 4). These results indicate that bringing more flexibility to the target has a significant effect on the decrease in LD signal. For example, the signal change induced by the largest strand, **AMPR-Target 3**, was less than 20% compared to **AMPR-Target 1**. Interestingly, the fact that the intermediate **AMPR-Target 2** gives the largest change in signal suggests that while misalignment increases with the introduction of a smaller non-binding region (10 nucleotides), a larger region (20 nucleotides) allows the bound phages to re-orientate in flow. While clearly requiring some further optimisation, the observed sensitivity of the technique shows promise for discriminating between DNA sequences that differ through base insertions or deletions alone (*e.g.* triplet repeats).^13^

### TEM studies

**Fig. 5:**
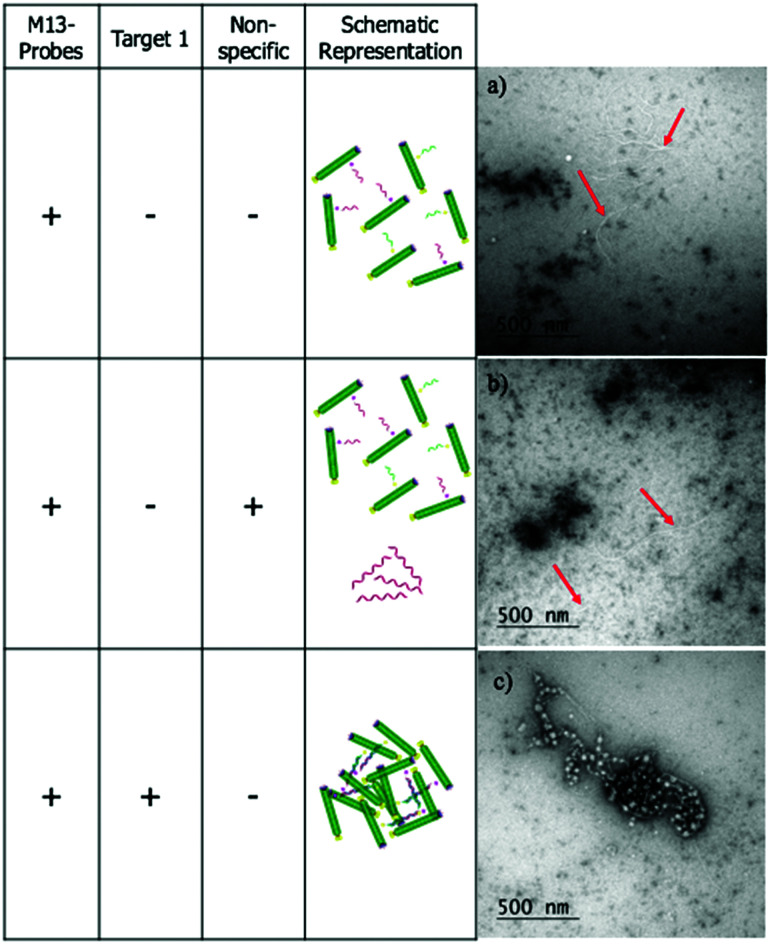
(a) M13 probes **AMPR-P1** and **AMPR-P2** alone, (b) M13 probes **AMPR-P1** and **AMPR-P2** plus **AMPR-NC**. (c) M13 probes **AMPR-P1** and **AMPR-P2** plus **AMPR-Target 1**. The dark clumps on the grids are indicative of uranyl acetate precipitation, caused by residual buffer salts within the sample. Red arrows indicate the location of the M13 probes in (a) and (b). In (c) the probes appear to be largely clumped together and wrapped up.

The LD studies indicated that the formation in solution of aggregated macromolecular structures, triggered by binding to complementary DNA, was responsible for the sensing signal. To investigate this further, negative stain transmission electron microscopy (TEM) performed on a copper nanogrid was undertaken on the *ampR* bacterial system to visualise this process (see ESI† for further details). These showed that in the absence of **AMPR-Target 1**, the M13-probes were evenly dispersed across the grid (Fig. 5a). Furthermore, in the presence of non-complementary DNA, no significant changes were observed (Fig. 5b). However, in the presence of the complementary target, a very different image was observed, with clustered macromolecular structures involving the M13-probe clearly visible (Fig. 5c). The formation of these large structures in solution would be expected to disrupt the fluid flow alignment of the bacteriophage, giving rise to a reduction in the LD signal.

### Sensing of pathogenic plasmid dsDNA

**Fig. 6:**
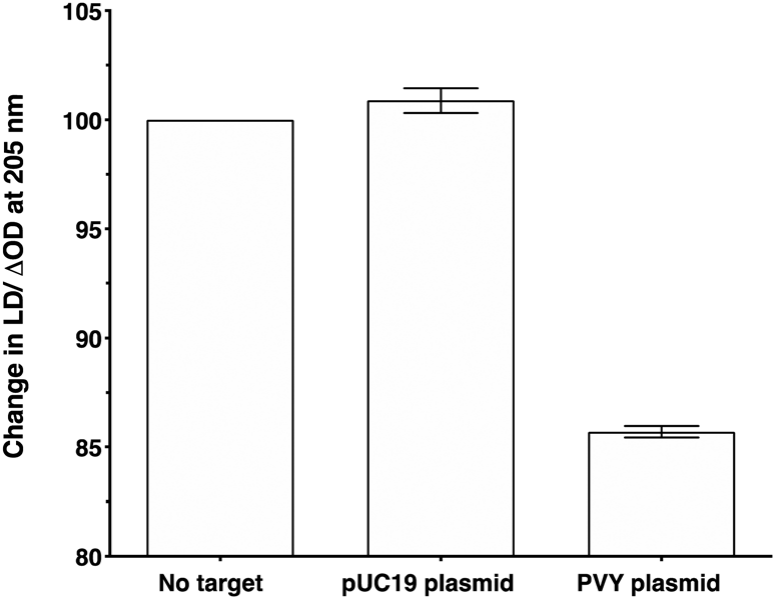
Percentage change in the LD signal (normalised to phage-alone) at 205 nm for M13-probe system **PVY-P1** and **PVY-P2** (*ca.* 1 nM) without target, after the addition of a control pUC19 plasmid (no decay in signal) and PVY plasmid (decay in signal). The concentration of both plasmids was 30 pM, 100 mM potassium phosphate buffer, 150 nM NaCl, at pH 7.2, RT, sample volume 100 μL. Data shows *n* = 3 (SEM).

To make the sandwich assay more relevant to the detection of raw target samples (*i.e.* closer to those actually tested in the field), a double stranded DNA (dsDNA) target was required. To test the hypothesis that dsDNA could also be detected by the LD probe system, a plasmid containing the same PVY coat protein gene was chosen (see ESI† for more details).^14^ The addition of probe system **PVY-P1** and **PVY-P2** to the plasmid sample revealed that LD detection was possible at a concentration of 30 pM (see Fig. 6). A control plasmid pUC19, which did not contain the PVY coat protein sequence, gave no reduction in LD signal under the same conditions.

## Conclusions

In previous work we demonstrated that the M13-LD sensing platform provides a novel approach to antibody diagnostics, with successful pathogen detection in less than one minute.^7^ We then went on to show how it could be adapted for gene sensing.^8^ In this paper we have developed our methodology further to demonstrate how DNA strand detection using LD spectroscopy can provide a viable alternative to more established read-out methodologies.^2–5^ Our approach, using two M13 probes in a sandwich assay, shows the versatility and sensitivity of the LD technique in detecting DNA target strands selectively and rapidly. It also showcases the ready use of a naturally occurring nanoparticle^15^ as a cheap alternative to the myriad of synthetic nanoparticles currently used within various DNA technologies.^16^ In achieving DNA detection down to picomolar levels, we envisage future LD assays operating either at the end point of an amplification step or directly in cases where further amplification is not required. Initial developmental work on such aspects is currently being undertaken in partnership with industry. This has required the development of a prototype small format LD spectrometer which utilises extension flow and simplified LED detection without significantly reducing sensitivity.

We envisage that this LD technology will ultimately lead to the design of portable (*i.e.* hand-held) devices giving rapid multiplexed (*i.e.* >1 sample) and multimodal pathogen detection. Regarding these last two points, it is important to note that the successful demonstration of DNA detection using the M13 scaffold means that this method can be seamlessly integrated into existing equipment designed for immunological M13-LD assays,^7*a*^ offering the opportunity for novel multiplexed assays performed in parallel, *i.e.* immune and DNA based detection on the same sample at the same time. This could be exceptionally important in conditions such as COVID-19 where different testing modalities are required to determine the state of the disease.

## Conflicts of interest

There are no conflicts to declare.

## Supplementary Material

CB-001-D0CB00135J-s001
