# Supplementary material for: Combining bacteriophage engineering and linear dichroism spectroscopy to produce a DNA hybridisation assay
Source: RSC Chem Biol. 2020 Oct 23;1(5):449–54. doi: 10.1039/d0cb00135j (PMC8341927; doi:10.1039/d0cb00135j)
Supplement: CB-001-D0CB00135J-s001 [file CB-001-D0CB00135J-s001.pdf]

Electronic Supplementary Material (ESI) for RSC Chemical Biology.
This journal is © The Royal Society of Chemistry 2020

Supplementary Information

Combining Bacteriophage Engineering and Linear Dichroism Spectroscopy to Produce a DNA Hybridisation Assay

Aysha Ali, Haydn A. Little, Jake G. Carter, Craig Douglas, Matthew R. Hicks, David M. Kenyon, Christophe Lacomme, Richard T. Logan, Timothy R. Dafforn and James H. R. Tucker

Contents:

1. Modified DNA strand synthesis and characterization Page S1
2. M13-Probe synthesis and characterisation Page S6
3. DNA strand binding studies Page S8
   3.1 UV/vis studies – T_m values
   3.2 Fluorescence Anisotropy Studies
4. LD studies Page S9
   4.1 Effect of conjugation on LD Alignment
   4.2 LD Sensing
      4.21 PVY target – single strand studies
      4.22 PVY target – control studies
      4.23 PVY target - plasmid studies:
   4.3 M13 Bacteriophage thermal stability studies
      4.31 wtM13 phage stability
      4.32 Thermal cycling – reversible binding
5 TEM studies Page S13

1. Modified DNA strand synthesis and characterisation

The modified strands were either bought from commercial suppliers or prepared in-house using standard solid-phase automated chemical synthesis. Relevant characterization details (MS and HPLC data) for the modified strands are shown below in Table S1 and in Figures S1-S3. Strand **AMPR-P1** conjugated to M13 was reported previously.[1] The modifications abbreviated in the table are shown below.

Oligo	Sequence (5'-3')	Calculated Mass	Observed Mass
**AMPR-P1 (no M13)**	`R-S-(TAMRA-dT)-ATG AGT ATT CAA CAT TTC`	6783	6783
**AMPR-P2 (no M13)**	`GCC TCA CTG ATT AAG CAT TGG-(6-FAM)-SSR'`	7323	7323
**PVY-P1 (no M13)**	`R-S-(6-FAM)-TTT TTT TTT GAA AAT GGA ACC`	purchased	purchased
**PVY-P2 (no M13)**	`TCG CCA AAT GTC ATT TTT TTT T-(6-FAM)-SSR'`	7554	7554

Table S1 – Calculated and observed masses of the synthesised oligonucleotides (ESI, negative mode).

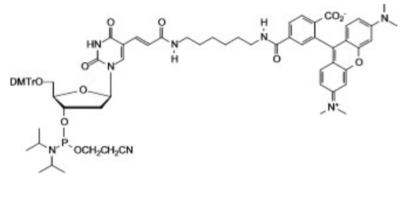

TAMRA-dT

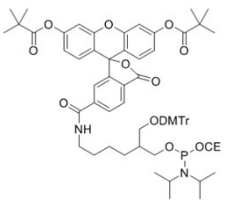

6-FAM

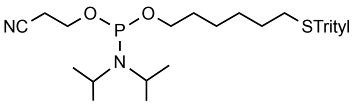

R-S-

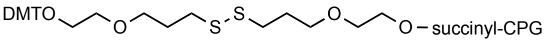

-SS-R'

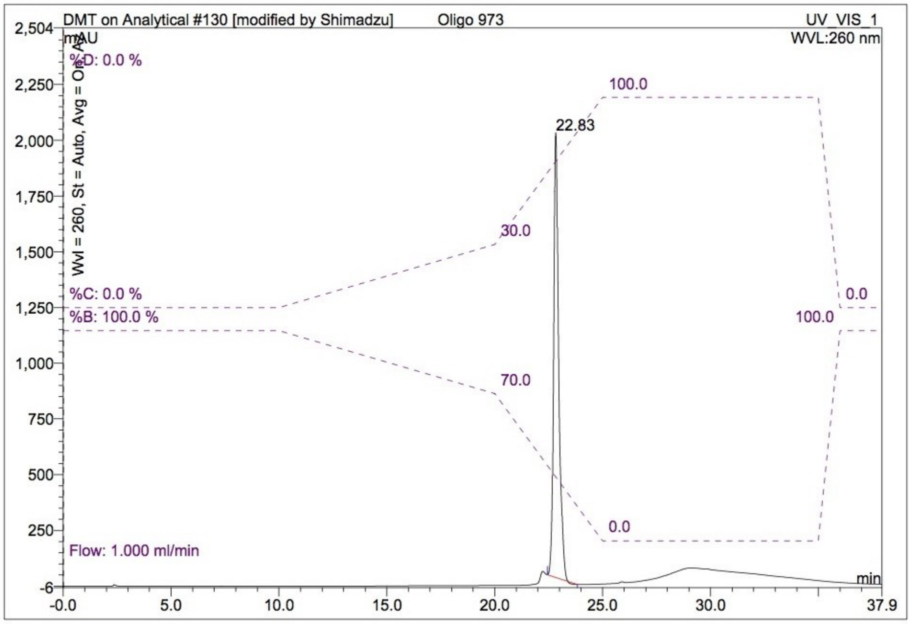

No.	Ret.Time min	Peak Name	Height mAU	Area mAU*min	Rel.Area %	Amount	Type
1	22.83	n.a.	1994.451	540.674	100.00	n.a.	BMB*
Total:			1994.451	540.674	100.00	0.000	

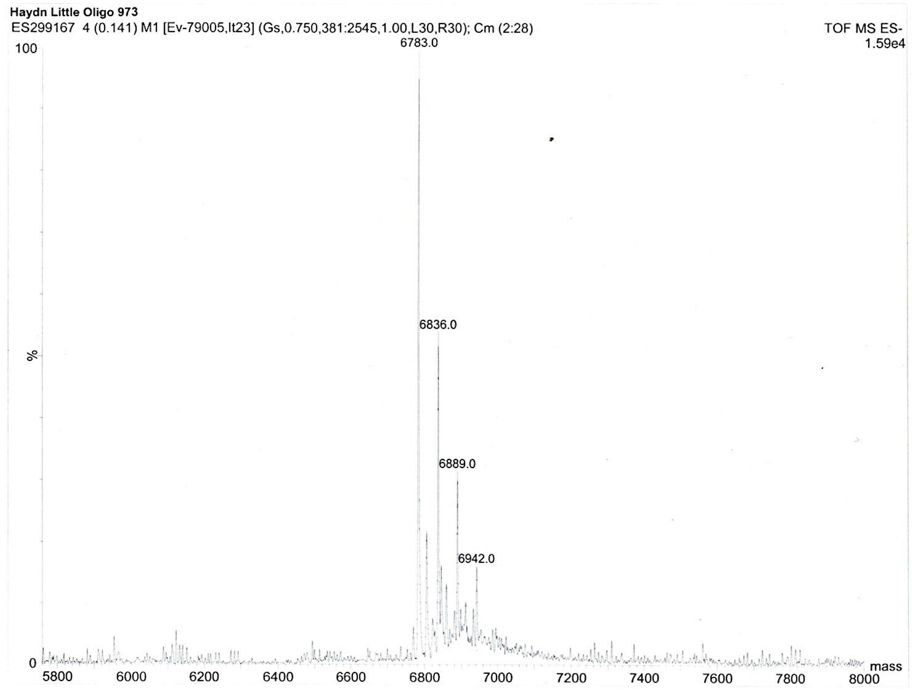

Figure S1. a) Analytical HPLC trace, monitored at 260 nm, and b) ESI MS of the oligo **AMPR-P1 (no M13)**.

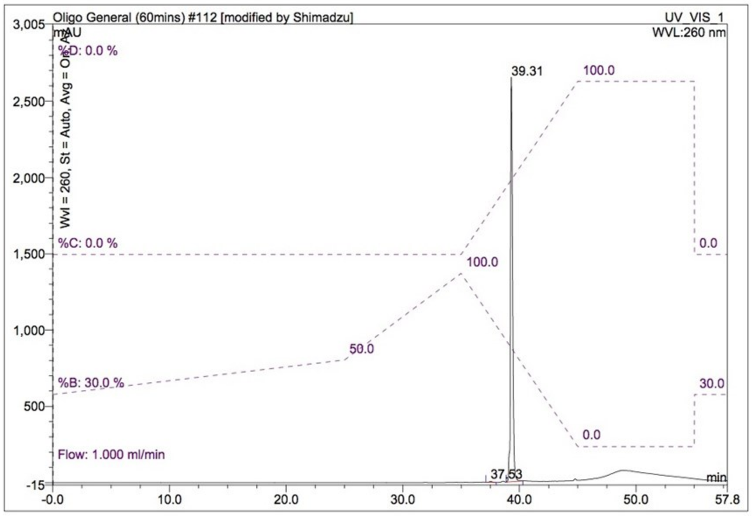

No.	Ret.Time min	Peak Name	Height mAU	Area mAU*min	Rel.Area %	Amount	Type
1	37.53	n.a.	6.104	1.613	0.29	n.a.	BMB
2	39.31	n.a.	2649.137	553.449	99.71	n.a.	BMB
Total:			2655.241	555.061	100.00	0.000	

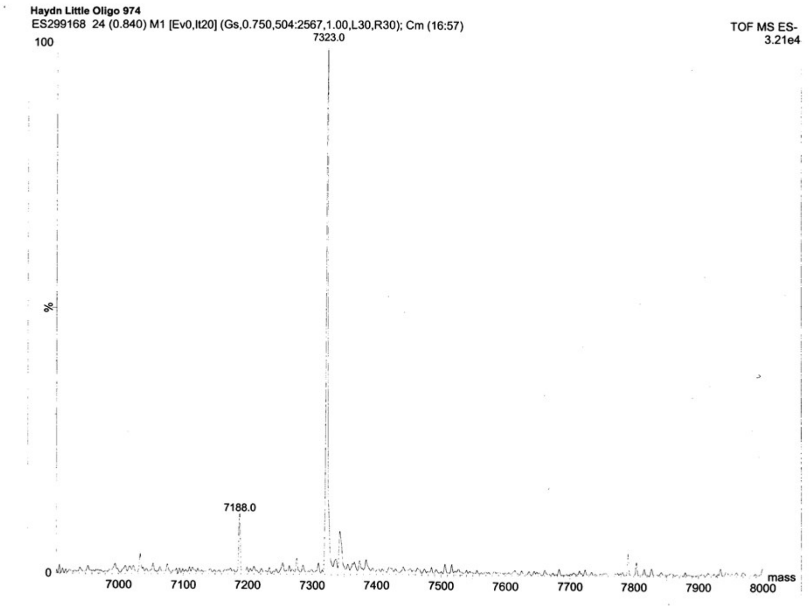

Figure S2. a) Analytical HPLC trace, monitored at 260 nm, and b) ESI MS of the oligo **AMPR-P2 (no M13)**.

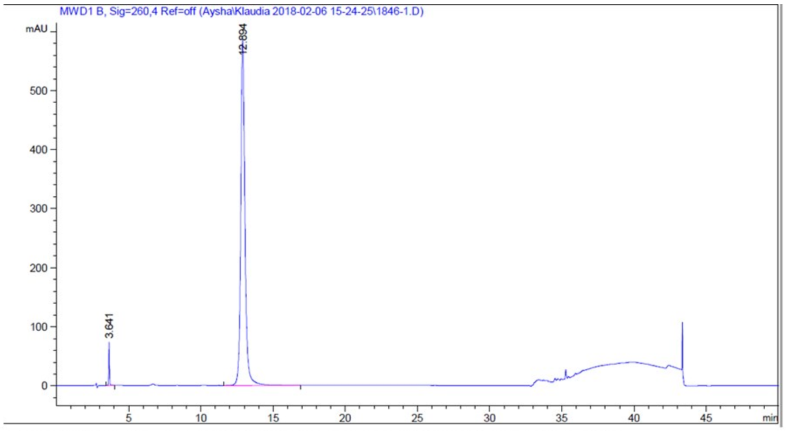


```
=====================================================================
                         Area Percent Report
=====================================================================

Sorted By             :      Signal
Multiplier            :      1.0000
Dilution              :      1.0000
Do not use Multiplier & Dilution Factor with ISTDs

Peak RetTime Type  Width     Area      Height     Area
  #   [min]        [min]   [mAU*s]     [mAU]       %
----|-------|----|-------|----------|----------|--------|
   1   3.641 BB    0.0553  260.95428   73.67761   2.1439
   2  12.894 BB    0.3056 1.19113e4   584.74969  97.8561

Totals :                   1.21722e4   658.42731
```

Base Peak Mass (Da)	Intensity	Spectral Quality
7553.5	2.55E+005	ok

Figure S3. a) Analytical HPLC trace, monitored at 260 nm, and b) ESI MS analysis of the oligo **PVY-P2 (no M13)**.

2. M13-Probe synthesis and characterisation

A general procedure for M13 bacteriophage isolation from bacterial culture is described below:

The inoculation of the bacterial culture in which M13 (New England Biolabs, UK) is produced was carried out in 400 mL of 25 g/L NB2 (Oxoid) solution. This was inoculated with 400 µL of 5 mg/mL of tetracycline dissolved in ethanol and all inoculation procedures were under sterile conditions. A 500 µL solution of One Shot Top10F' *E. coli* (F' [*lac*I^q, *Tn*10(Tet^R]) (Invitrogen, California, USA) and 0.04 mg of M13 bacteriophage in 50 mM potassium phosphate (at pH 8.0) was added to the same flask. The culture was then incubated for 16 hours, at 200 rpm, 37 °C in an orbital shaker. After the 16 hour incubation the culture was sedimented (Avanti J-24, Beckman Coulter USA centrifuge with a JLA 10.5 rotor) at 10000 g for 20 minutes at 4 °C. To ensure all the *E. coli* had been removed the supernatant is taken and the same sedimentation step was repeated. After the second sedimentation step 80% of the supernatant was then taken from the tubes and added to a beaker. The M13 was precipitated using a 2.5% w/v solution of polyethylene glycol (PEG) 6000 and 2.5 M NaCl (5:1 ratio of PEG 6000/supernatant). The solution was left to stir at 4 °C for 1 hour. The precipitated solution was sedimented as above but for 25 minutes instead of 20 minutes. After the supernatant had been discarded the pellet was resuspended in 1 mL of 50 mM potassium phosphate buffer at pH 8.0 in a microcentrifuge tube. To remove any remaining *E. coli* cells in the solution the tubes were sedimented for a further 5 mins at 14000 rpm (FX241.5p rotor, Microfuge® 16, Beckman Coulter, USA) and the pellet was discarded. Finally, the PEG/NaCl precipitation was repeated at a ratio of 5:1 PEG 6000/supernatant. The resulting solution was sedimented in a bench top centrifuge (FX241.5p rotor, Microfuge® 16, Beckman Coulter, USA) at 14000 rpm for 15 minutes. The resulting supernatant was discarded, and the pellet was resuspended in 200 µL of 50 mM potassium phosphate buffer, pH 8.0. The M13 sample was stored at 4 °C until use.

The two-step conjugation procedure to DNA was performed as described previously (see Scheme S1),[1] involving a reaction with each of the four DNA strands listed in Table S1 (as their thiols, stored in TCEP).

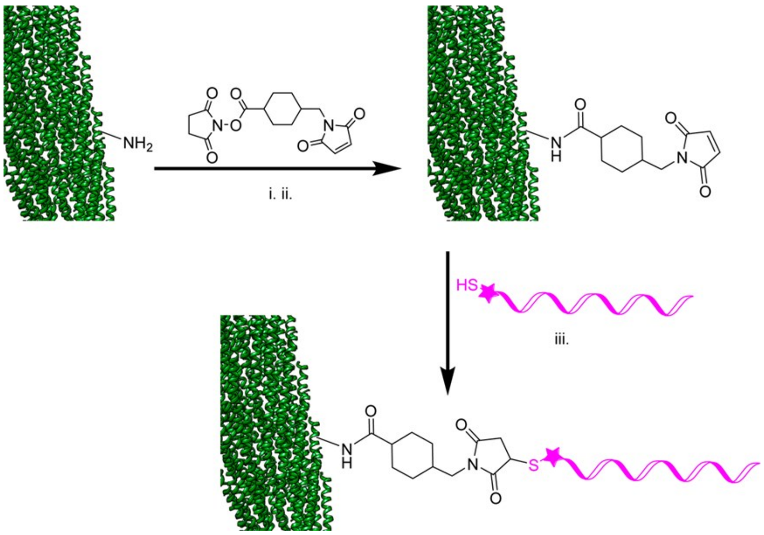

Scheme S1. Conditions: (i) SMCC in DMSO, 1 h, RT. (ii) glycine, 15 min, RT. (iii) Reaction with the thiol in its reduced thiol form, 16 h, 5 °C (scheme taken from reference [1])

Once the bioconjugation procedure had been carried out, the M13-probe conjugates were purified to remove any DNA probes that had not undergone conjugation. Purification was carried out using size exclusion chromatography (SEC).

During the elution procedure, three different wavelengths were monitored to determine which peaks contained the M13-probe conjugates and which contained any unreacted DNA or small molecules: The 269 nm signal provided information on the M13 bacteriophage (the protein capsid), the peak at 260 nm denoted DNA from both the virus and probes and the one at 495 nm corresponded to the fluorescein tag on the oligonucleotide probe. An example trace for the probe **PVY-P1** is shown in Figure S4. After their synthesis, all M13-Probes were stored in 150 mM NaCl, 100 mM potassium phosphate buffer, pH 7.2.

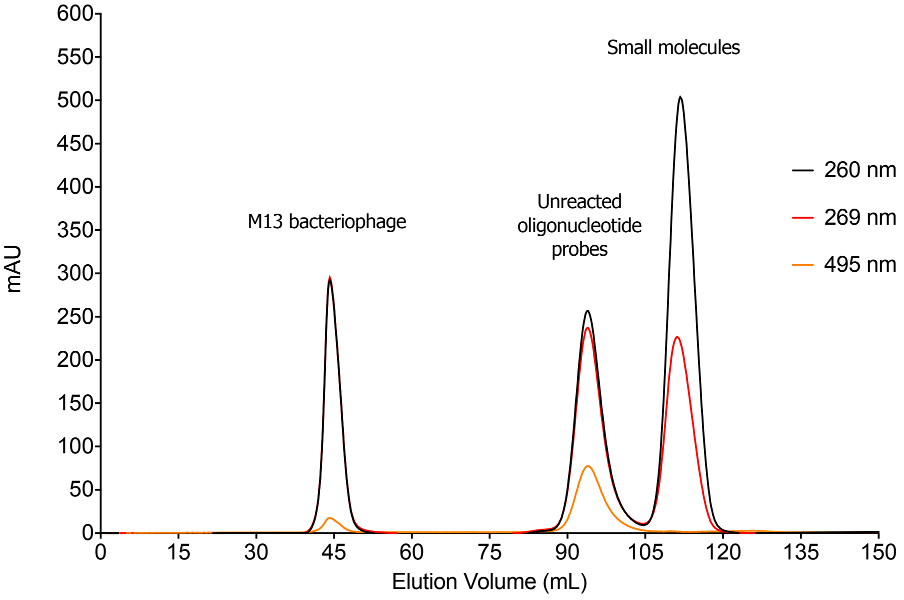

Figure S4. An example of the size exclusion chromatogram produced during the purification of the M13-phage after conjugation to **PVY-P1**. All samples were eluted into 100 mM potassium phosphate buffer, 150 nM NaCl, at pH 7.2, at RT.

3. DNA strand binding studies

3.1 UV/vis studies – T_m values

Table S2 shows the experimental T_m values recorded using UV-vis spectroscopy for the AMPR system probes (before conjugation to M13). The observed T_m values were similar to calculated values. **AMPR-Target 1** bound **AMPR-P1** and **AMPR-P2** together, showing only one melting curve and thus one melting temperature. The value of 64.5 °C was only ca. 6.5 °C lower than the value for **AMPR-Target 1** with an intact, complementary 39-mer sequence of the P1 and P2 probes combined.

Table S2. Melting temperature values for **AMPR-Target 1** with **AMPR-P1** and/or **AMPR-P2**. 2.5 µM of each strand, in 10 mM sodium phosphate buffer, 100 mM NaCl, pH 7.2.

Strand combination studied	Observed T_m °C*
**AMPR-P1** plus **AMPR-Target 1**	51.5 (± 0.5)
**AMPR-P2** plus **AMPR-Target 1**	63.5 (± 0.5)
**AMPR-Target 1** plus complementary 39-mer sequence	71.0 (± 0.5)
**AMPR-Target 1** plus **AMPR-P1** & **AMPR-P2**	64.5 (± 0.5)

*Change in hyperchromicity at 260 nm; three experimental repeats for SEM to the nearest 0.1 °C. Error in instrument data = ± 0.5 °C.

3.2 Fluorescence Anisotropy Studies

Figure S5 shows the results for the probes alone (no M13 attached) for the AMPR system hybridizing to their complementary target **AMPR-Target 1**. Similar trends were observed for the M13 conjugated probes (see main text), albeit with lower FA intensities for the conjugated system. The non-complementary DNA targets again showed no interactions with the probe strands.

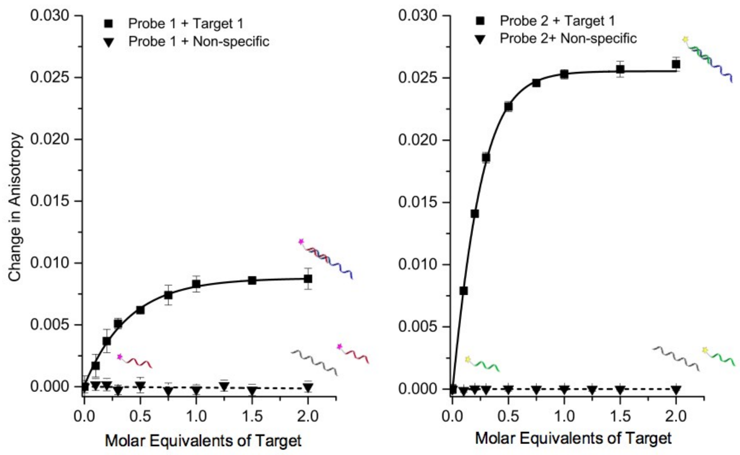

Figure S5. Fluorescence anisotropy changes of **AMPR-P1 (no M13)** and **AMPR-P2 (no M13)** upon the addition of **AMPR-Target 1** and non-specific DNA **(AMPR-NC)**. 20.5 nM M13-Probe ½, 150 mM NaCl, 100 mM potassium phosphate buffer, pH 7.2. Data shows $n = 3$ (SEM).

4. LD studies

4.1 Effect of conjugation on LD Alignment

The LD spectra of unreacted wild-type phage (wtM13) was compared to that of the conjugated phages, **AMPR-P1** and **AMPR-P2**, as shown in Figure S6. Modification caused a small decrease in signal, which was deemed acceptable for sensing purposes. No difference in LD peak intensity was observed between the two probes.

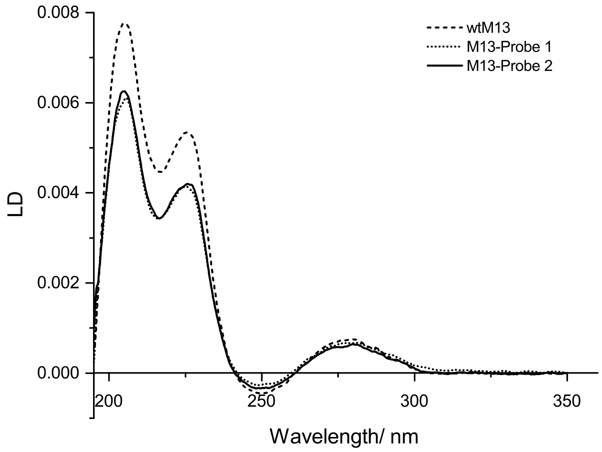

Figure S6. Comparison of wtM13 alignment (dashed) to **AMPR-P1** (M13-Probe 1, dotted) and **AMPR-P2** (M13-Probe 2, solid), each modified with 15 oligonucleotides on average. All bacteriophage concentrations at 0.046 mg mL^{-1} as determined by absorbance spectroscopy at 269 nm (E^{269nm} - 3.84 cm^2mg^{-1}) performed in 100 mM potassium phosphate buffer, 150 mM NaCl, pH 7.2.

4.2 LD Sensing

4.21 PVY target – single strand studies

To determine whether **PVY-Target 1** could be detected using LD spectroscopy, aliquots of target DNA were added to the phage system **PVY-P1/PVY-P2** in the low nM range and the change in LD signal was monitored after each addition (sample volume kept constant throughout at 100 µL). A decrease in LD signal was observed with increasing concentrations of **PVY-Target 1**, with the lowest concentration of target that could be detected being 0.05 nM (or 50 pM, second data point in Figure S7).

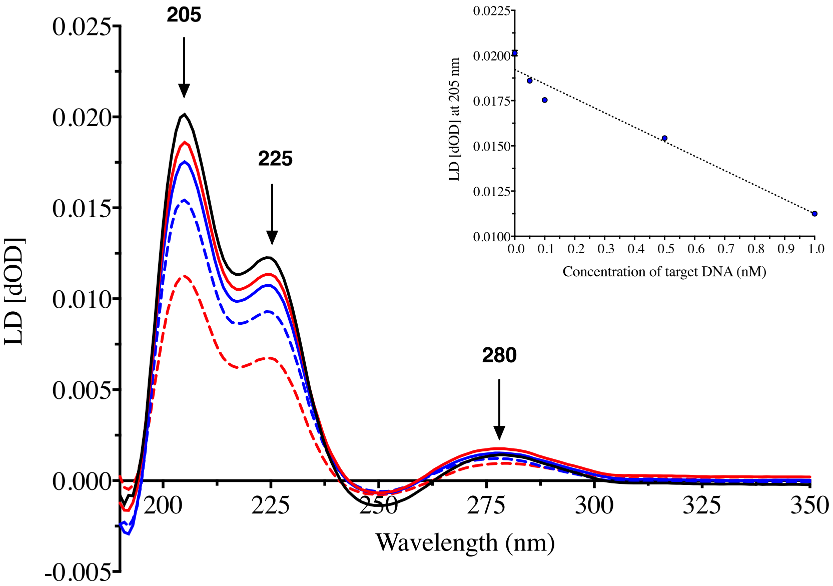

Figure S7. LD spectrum after the addition of increasing amounts, up to 1 nM, of **PVY-Target 1** to **PVY-P1** and **PVY-P2**. The inset shows the change in LD signal at 205 nm plotted against the concentrations of **PVY-Target 1** used for the limit of detection (LOD) studies. The samples (each 100 μL) were in 100 mM potassium phosphate buffer, 150 nM NaCl, at pH 7.2, at RT. Data in the inset shows $n = 3$ (SEM).

4.22 PVY target – control studies

To confirm any decay in LD signal was due to the M13-probe system binding to the PVY target, and not any non-specific interactions, control studies were undertaken. The results showed that firstly no decay in LD signal when an equimolar amount of either the PVY target or the non-complementary control was added to WT-M13 (Figure S8A). A similar result was seen when the non-complementary control was added to the PVY M13-probe system (Figure S8B).

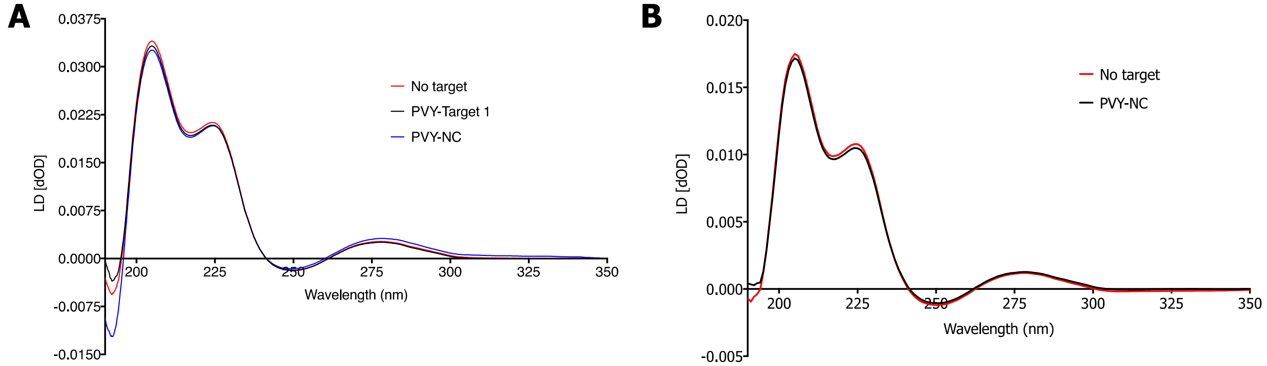

Figure S8. A) LD spectra of **WT-M13** (red) with **PVY-Target 1** (black) and **PVY-NC** (blue); **B)**. LD spectra of **PVY-P1** and **PVY-P2** with no target (red) and **PVY-NC** (black). The samples were at 1 nM in 100 mM potassium phosphate buffer, 150 nM NaCl, pH 7.2 at RT.

4.23 PVY target - plasmid studies

To detect the plasmid samples the plasmid DNA was heated to 85 °C, as any higher would compromise the M13 bacteriophage, for 30 seconds to allow for double stranded DNA denaturation. The phage probe system (ca. 1 nM in each probe, **PVY-P1** and **PVY-P2**) was then immediately added to the plasmid sample, with the solution left to cool to room temperature to allow for the probes to anneal to the denatured plasmid sample.

4.3 M13 Bacteriophage thermal stability studies

This section describes firstly, an examination of the thermal stability of the M13 bacteriophage to determine what effect increasing the temperature has on the nanostructure. Secondly, it shows a thermally reversible binding model for the detection of Target 1 and the regeneration of the free M13-Probes.

4.31 wtM13 phage stability

A melting study was performed by monitoring the LD spectra while increasing the temperature of the M13. The data in Figure S9 shows that wtM13 is stable up to 80 °C before losing much of its LD alignment, and is completely denatured by 90 °C. These data are consistent with the previous studies of viral-PCR conducted by Carr-Smith *et al.*[1]

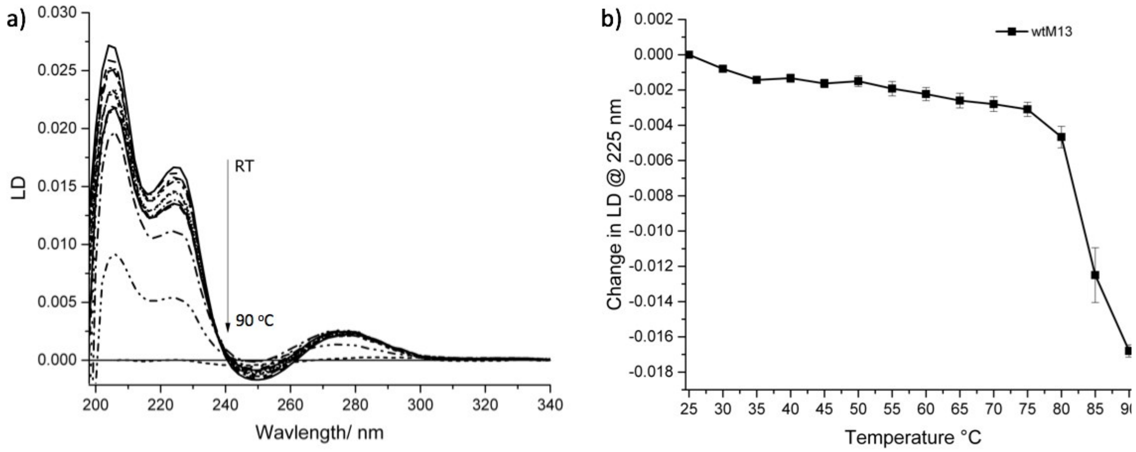

Figure S9. a) wtM13 stability and effect on LD with increasing temperature. With increasing temperature there is a decrease in LD signal intensities. b) This effect is more apparent on investigation of the change in LD at a single wavelength vs temperature; 225 nm (squares), gives information on the alignments of the pVIII peptide backbone and thus the alignment of the M13. Temperature changes at a rate of 1.0 °C per min with a 5 min equilibration time at each temperature. Measurements were taken every 5 °C. Sample was prepared in 50 mM NaCl, 100 mM potassium phosphate buffer, 0.1% TWEEN 20, pH 7.2. wtM13 concentration = 0.046 mg mL^{-1}.

4.32 Thermal cycling – reversible binding

An VT LD experiment was set up to monitor the stability of the M13-Probes (AMPR system) through a series of DNA melting and annealing steps. The heating profile (Figure S10) is indicated by coloured triangles: DNA melting 25 °C - 65 °C (red), DNA annealing 65 °C - 50 °C (blue), second DNA melting 50 °C - 65 °C followed by further heating to 90 °C (red). Before the addition of **AMPR-Target 1**, an LD measurement of mixed M13 probes **AMPR-P1** and **AMPR-P2** (labelled M13-Probes) was performed. The target was then added at one molar equivalent to the probe strands and as expected, a decrease in LD was observed (blue dotted line). The results indicate the reversibility of the system over two cycles by heating to ca. the T_m value of the duplex, which regenerates the signal, followed by cooling. Heating to 90 ^{o}C denatures the M13 bacteriophages, resulting in a larger loss of LD signal.

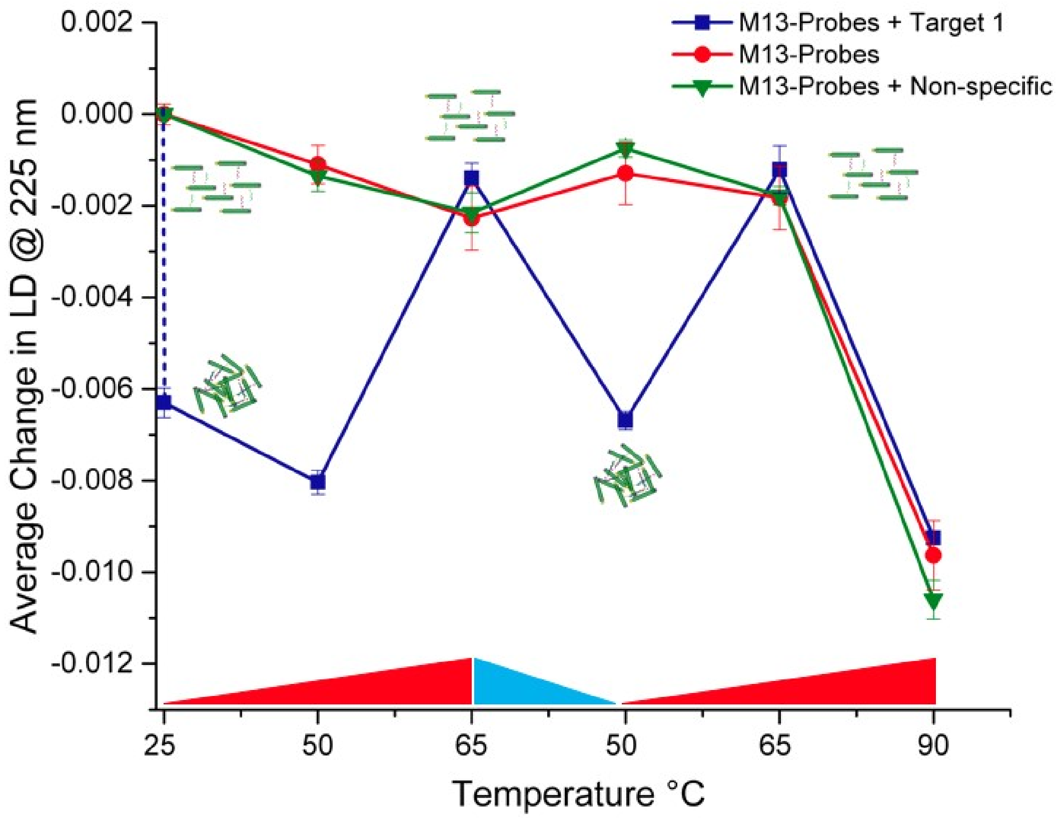

Figure S10. Thermal reversion using **AMPR-P1**, **AMPR-P2** at 1.4 nM each and **AMPR-Target 1** (blue squares) and associated control studies (either no target or non-specific, **AMPR-NC**). All samples were prepared in 50 mM NaCl, 100 mM potassium phosphate buffer, 0.1% TWEEN 20, pH 7.2. Blue dotted line indicates that an initial alignment measurement was taken before the addition of the target. Heating profile is indicated by coloured triangles at the bottom of the graph. Temperature changes at a rate of 1.0 °C per min with a 5 min equilibration time at each temperature. Y axis values are different to those in main text due to a different machine and Couette cell being used. Measurements were taken every 5 °C. Data shows $n = 3$ (SEM).

5. **Transmission Electron Microscopy (TEM)**

Preparation of Carbon-coated Grids - Formvar/Carbon on 200 Mesh Copper (Agar Scientific, UK) were treated by glow discharge. Grids are placed carbon side up on a glass slide into a E200 Autosputter coater for 20 seconds at 10 mA.

Staining and Visualisation – the M13 samples (5 μL of 0.04 mg/mL) were added onto the carbon coated grid. The liquid was removed by wicking with filter paper after 60 seconds. Then 1% uranyl acetate (5 μL) was added and wicked off with filter paper after 60 seconds. The grids were allowed to dry for 5 minutes. A JEM-2200FS TEM (Joel, Japan) was used to view the grids at 8000X, 10000X, 12000X and 15000X magnification.

Reference:

[1] J. Carr-Smith, R. Pacheco-Goḿez, H. A. Little, M. R. Hicks,S. Sandhu, N. Steinke, D. J. Smith, A. Rodger, S. A. Goodchild, R.A. Lukaszewski, J. H. R. Tucker and T. R. Dafforn, *ACS Synth. Biol*,. 2015, **4**, 1316−1325.
